# Older Persons' Experiences of Depressive Ill-Health and Family Support

**DOI:** 10.1155/2013/837529

**Published:** 2013-09-01

**Authors:** Anne Lyberg, Anne Lise Holm, Erna Lassenius, Ingela Berggren, Elisabeth Severinsson

**Affiliations:** ^1^Centre for Women's, Family and Child Health, Faculty of Health Sciences, Vestfold University College, P.O. Box 2243, 3101 Tønsberg, Norway; ^2^Department for Nursing, Health and Culture, University West, 461 86 Trollhättan, Sweden

## Abstract

The aim of this study was to explore experiences of the meaning of family support among older persons with depressive ill-health. Data were collected from twenty-nine participants through semistructured interviews and analysed using interpretative hermeneutic and reflective methodology. The findings revealed a main theme, hovering between feelings of belongingness and aloneness in relationships with family members, based on two themes: a sense of being worthy and a sense of being unworthy. Experiences of support and lack of support from family members were not opposites but connected in internal relationships and can be pictured as a movement on a continuum of ambiguity. Family support promotes the emotional needs of older persons with depressive ill-health to be confirmed. The family plays a vital role, not always by direct assistance, but indirectly by supporting the older person's own “guiding principles” for managing her/his situation. The feelings of aloneness as well as shame and guilt at poor or absent family responsiveness should be adequately addressed. Innovative nursing care can lead to improvement by focusing on acquiescence to the older person's life situation.

## 1. Introduction

Depressive ill-health is highly prevalent in the general population and constitutes a major public health problem  [1]. Depression is considered one of the most common disorders among community dwelling older adults with a prevalence of between 12 and 20%  [2]. An older-age related increase in depression has been documented  [3]. Although older people constitute a large proportion of the population, older persons with mental ill-health have been neglected in terms of research as well as access to mental health services, and therefore knowledge of older persons' experiences of their health situation is generally poor  [4].

Deinstitutionalization of mental health services and the promotion of initiatives whereby people are supported to live in their own homes are strategies in many western countries  [5]. One reason for this political strategy is the fact that personal independence and freedom are human rights in western society  [6, 7]. Nevertheless, freedom and independence vary and can be positive as well as negative. For older persons suffering from depressive ill-health, the potential need for family members to provide care and support might lead to the feeling of loss of independence for both the older person and the family. According to Martinsson et al. [8], older persons suffering from depressive ill-health constitute a vulnerable group. However, depression in older persons is a complex health problem due to the difficulty of distinguishing between depression and symptoms of physical ill-health, dementia, normal aging, and grief  [9]. Several studies have demonstrated a significant association between depressive symptomatology and somatic diseases [10]. Poor somatic and mental health may exert a considerable psychosocial and physical burden on an individual and her/his family. 

The perceived availability of support from others can serve as a buffer against the development of a depressive episode [11]. Positive family relationships can improve mental ill-health, while support from family members often facilitates recovery [12]. In contrast, low levels of support have long been associated with the onset of depressive symptoms, delayed recovery, and a tendency towards chronic depression [13]. In this study, family support is defined as both structural and functional. The former refers to the level and frequency of contact, while the latter includes emotional, tangible, informational, and appraisal support [14]. Emotional support is reported to be the most important because it has been clearly linked to health in terms of direct and buffering effects [15]. It includes feelings of trust, concern, empathy, and love from others [14] and can be derived from a variety of relationships, although Ahlström et al. [12] found that alleviation of depression was more associated with perceived family support than friend support. However, Nasser and Overholser [16] consider that relationships with friends can be more effective than those with family members for alleviating depressive illness. One explanation could be that a depressed family member may believe that others in the family feel obliged to maintain relationships and provide support, while friends remain supportive out of friendliness and companionship. Family relationships may not always be good for a depressed person's health, as disagreements within the family may be associated with the onset of symptoms and therefore destructive [16]. In general, family relationships can have negative and stressful components as well as helpful aspects. It is also likely that depression can reduce an individual's access to support. Depression is often accompanied by considerable social isolation and withdrawal from activities that are essential for providing emotional support and assistance [17]. In addition, Martinsson et al. [8] highlighted some difficult existential components of old age such as the struggle against futility, decrepitude, and invisibility.

Greater understanding of the meaning of family support can guide caregivers in their work with older persons who suffer from depressive ill-health and their family members. It might inspire them to avoid too much focus on diagnoses and symptoms and instead acquiesce to the old person's life situation. The aim of the study was to explore experiences of the meaning of family support among older persons with depressive ill-health.

## 2. Methods 

This study has an exploratory and descriptive design [18] based on interviews [19]. The authors used Gadamer's [20] philosophical existential hermeneutics as the overall methodological approach. Hermeneutic interpretation is appropriate for describing human beings' existential experiences based on feelings, inner thoughts, and beliefs about life. Gadamer's philosophic and existential hermeneutics is more than a data interpretation method. In the context of long-term depressive ill-health in later life and perceptions of family support, it is both an underlying philosophy and a specific mode of interpretation. Interpretation is necessary as it is impossible to obtain direct or complete access to the old person's mind when searching for the meaning of experiences. 

### 2.1. Recruitment and Participants

The inclusion criteria were persons aged over 60 years who had been diagnosed with a long-term depressive or mood disorder, the ability to understand and speak the Norwegian language, living in their own homes and receiving ongoing formal support from primary health care or mental health care during the previous six months. When the interviews were conducted none of the participants were hospitalized. Twenty-nine persons were recruited by nurse managers and/or their contact person in the health care system on the west coast and the south-eastern part of Norway. Twenty-six women and three men aged between 60 and 91 years (mean 66 years) were interviewed. Five were married and/or living with their partners, thirteen were widows, nine were divorced, and two had always lived alone. All of the participants had children. Most had suffered from their mental health problems for many years, a considerable number from early in their adult life. Only one of the participants below retirement age was in employment; the others were housewives or received disability benefit. For most of the participants, the experience of stressful life events was obvious. For instance, eight had tried to commit suicide, while nine had children who had committed suicide or died as a result of accidents and drug abuse. The participants were in good physical health for their age. Only one man linked his depressive ill-health with a previous stroke and the symptoms it had caused. 

### 2.2. Data Collection

Data were collected between August and December 2011 by means of semistructured interviews. Each of the interviews lasted approximately one to two hours and, depending on what was most comfortable for the participants, took place in their own homes, mental health centres, or the researcher's office. The participants were encouraged to have a dialogue  [20] about their experiences of depressive ill-health focusing on family support. The opening question was “Can you please describe your experiences of help and support from your family?” They started narrating how depression had influenced their lives for many years, describing the consequences for themselves and their family relationships. The interviews were conducted, audiotaped and transcribed verbatim by two of the authors (Anne Lyberg and Anne Lise Holm). 

### 2.3. Data Analysis

The data were analysed through the process of interpretative hermeneutics inspired by Gadamer [20], and reflective methodology in accordance with Alvesson and Sköldberg [21] was employed. In this study, interpretation is considered to take relevant information into account, using one's humanity to gain a perspective on the world and understand others. Preunderstanding is a central component as it represents the historical and cultural horizons that influence interpretation, thereby constituting understanding [20]. Four of the authors are mental health nurses and therefore have theoretical and practical knowledge of depressive ill-health among older people. The fifth author is an intensive care nurse and thus not familiar with the mental health context. All the authors have long experience of conducting research in the field. Their preunderstanding was brought into the open and reflected upon during the research process.

The interpretation involved several phases. The transcribed interview texts were first read carefully by the first author (Anne Lyberg) and one of the authors (Erna Lassenius) who had not been involved in the data collection in order to achieve a naïve understanding of the lived experiences of the older people [21]. Here, naïve means ingenuous reading, trying to follow Martinsen's [22] advice about reading slowly and being sensitive and open to the underlying, intentional meaning of the text. The authors (Anne Lyberg, Erna Lassenius) had close contact and frequent discussions in order to share the meaning and expand their understanding of the experiences reported by the participants. It was a dialectical process that moved the hermeneutic circle from preunderstanding via interpretation to understanding, with a strong relationship between the parts and the whole, resulting in an initial interpretation with themes and subthemes. To achieve a comprehensive understanding of the meaning of the text, a second interpretation was made by all five authors in order to deepen the understanding. Critically reflective questions were put to the meaning units and theme areas. The purpose was to reach a higher level of abstraction and, as suggested by Gadamer [23], to find “hidden meanings” and avoid what Alvesson and Sköldberg [21] described as a risk of simple empiric summing up of surface structures. The authors attempted to make it possible for the text to express aspects of the meaning of family support from the perspective of older persons in the context of depressive ill-health. The second interpretation resulted in consensus about the themes and subthemes as well as the development of a main theme (Table 1). 

### 2.4. Ethical Considerations

The study was approved by the Regional Committee for Medical and Health Research Ethics of Western Norway (no. 2010/2242). Approval was also granted by the managers in the communities where the participants live and receive different kinds of mental health support and service. The ethical procedures specified by the Declaration of Helsinki  [24] were applied. All the participants were provided with oral and written information by a nurse manager and/or their contact person in the health care system, and those who agreed to participate signed a consent form to confirm their voluntary participation. In addition, they were assured that their identities would not be disclosed and informed of their right to withdraw from the study at any time. 

## 3. Results

The meaning of older persons' experiences of depressive ill-health and family support were interpreted as follows: *hovering between feelings of belongingness and aloneness in relationships with family members* isbased on two themes:* a sense of being worthy *and* a sense of being unworthy. *The theme of a sense of being worthy comprised two subthemes: *being part of the family *and *being able to live up to family expectations. *The theme of a sense of being unworthy contained three subthemes: *being a burden on the family, being unable to live up to family expectations, *and* feelings of guilt and shame.*

### 3.1. Hovering between Feelings of Belongingness and Aloneness in Relationships with Family Members

The comprehensive understanding is based on the interpreted dialectical process, which indicates that older persons who suffer from depressive ill-health experience belongingness versus aloneness. It can also be interpreted as a dialectical process between feelings of living up to and being unable to live up to demands resulting in a sense of being ignored by others or rejecting their own desires. The meaning of family support concerned the participants' feelings of making demands on themselves due to expectations from family members, leading to hovering between feelings of belongingness versus aloneness. 

### 3.2. A Sense of Being Worthy

The findings revealed that throughout life different elements of family relationships are a source of emotional and practical support. The family was extraordinarily significant in helping older persons to develop meaningful ways to manage their mental health difficulties and social situations. Many strongly desired to feel worthy and found the family a source of strength. Therefore, the theme of *a sense of being worthy* includes two subthemes: *being part of the family* and *being able to live up to family expectations*. 

#### 3.2.1. Being Part of the Family

The experience of having a family whose members are loyal and ready to lend a hand in a supportive manner seemed to strengthen the older persons. One woman stated the following:

**I have a feeling of getting back what I invested in my children when they were young. I am getting it back from them now and we are a family that cares for each other. **

This kind of strengthening support was described as closeness and responsive emotional backup from family members that did not undermine the older person's self-determination. There was a natural give and take. Despite the older person's obvious ill-health, family members could see when help was needed and respond accordingly. Another woman described an episode when she was alone in her holiday home and in despair due to fear of losing control and committing suicide.

**By coincidence my son came that very night and we had a long talk. He understood how serious it was. The next morning he knocked on my bedroom door and told me he had arranged for an appointment with the doctor that same day. That was a great help. I do not think I would have gone to the doctor on my own. You lose your perspective on things when you are depressed.**

The interpretation of the narratives revealed various examples of concealed and unspoken expectations pertaining to what is appropriate in family relationships. Whilst some had family members with whom they could unburden their heart and talk about their mental health problem, others found that their family avoided the issue. Sometimes the family relationship could also be a painful reminder of the other side of the situation. One woman told how she was unable to enjoy the companionship of the family when visiting her daughter who lived in another part of the country.

**I was stupid, I began to think about returning to my empty apartment while I was still with my daughter's family. I should not have done that. I should have enjoyed the days we were together.**

Being part of the family also included underlying fears of losing contact. When adult children were extremely busy in daily life, it was difficult for the parent to express her/his needs. The older family member could worry a great deal about others in the family and did her/his best to protect them. Nevertheless, the situation was not without bitterness, especially in relation to their children.

**I have a fantastic relationship with my children but I cannot tell them everything if we are going to stay on polite terms. I have become accustomed to the fact that our relationship must be on their terms, it is the same for all parents and grand-parents. You must appreciate what you have and I am very fond of them.**

In general, although they wanted to be included, the older persons had fairly low expectations of obtaining support, especially from their children. Some participants thought that the feeling of being part of the family would have been stronger if their parents were still alive and siblings were younger. The fact that siblings had also become older and suffered from various health problems limited the opportunity for company and support.

#### 3.2.2. Being Able to Live Up to Family Expectations

The older persons were often surprised that their family members had endured their mental health problems for so many years. Despite their own problems and lack of initiative, the older persons made an effort to try to help their family members. They experienced a need to improve their children's life situation, which was a continual source of guilt because they did not always have the strength due to depressive ill-health. One woman was proud of managing to provide help.

**I cook dinner when I visit so that it will be ready when they return from work. My grandson asks me to make his favourite meal and the smile he gives me when I do makes me so happy. I long for the next time I can visit them.**

### 3.3. A Sense of Being Unworthy

As expected, not everyone obtained family support, and what was initially experienced as strengthening increasingly developed into the opposite, namely, nonsupportive behaviour. Family relationships, especially when one is older, a parent and suffering from depressive ill-health, are not without contradictions. This demands a great deal of balancing as presented in the three subthemes: *being a burden on the family, being unable to live up to family expectations, *and *feelings of guilt and shame.*

#### 3.3.1. Being a Burden on the Family

The need for someone to talk to without being a burden was a constant concern for most of the older persons. However, being in need of support and not being a burden on the family often implies a contradiction between wishes and reality. Even when they experienced an immense need to talk to their family members, they tried to be careful in order to not abuse or overuse the family's accessibility. The older persons did not want their concerns to add to the burden of their family members. This contradiction seemed to create difficulties, a sense of loss, loneliness, and isolation in many relationships. When their longing for meaningful connection was not fulfilled, it could change into withdrawal from relationships with others. Nevertheless, the older persons were quite resolute that they did not want to burden their adult children with their own fears. They also considered that some of their inner thoughts were unsuitable for sharing with the family and many of the participants reported their need for more attention and supportive conversations with professionals. The relationship with the family was often characterised by one sidedness in that the older family member exhibited more understanding than she/he received in return. As the quotation below demonstrates, many parenting and shielding considerations are involved.

**My son and his family do not live very far away, but I rarely see them. You see, my son has a demanding job and has to do a lot of travelling so when he is home he wants to stay at home. That is understandable. In addition, he thinks that the health care system is responsible for supporting me. **

Living with mental baggage over the years can lead to a sense of internal splitting and increased ambivalence. 

#### 3.3.2. Being Unable to Live up to Family Expectations

Some of the participants assumed that the family had expectations of them, comprising a pressure to conform to “other” standards, although these were not always expressed directly. Whether or not this was actually the case, it made them feel less valuable. Being unable implies fear and anxiety; their abilities were inadequate to meet the expectations of family members. This caused deep desolation and distance which they coped with by telling themselves that their family did not understand them.

**My daughters are occupied with their jobs and all sorts of things. I think they expect me to be more helpful with looking after my grandchildren, but they do not realise how ill I am. We do not have conflicts, they know where to find me if they want. If I am not worthy of their attention I will manage on my own. **

There was also a nagging sorrow about the loss of their previous life before the development of depressive ill-health. If family members do not see or confirm their situation, it can make them doubt their own value as a person. One man deeply mourned the loss of his wife and work due to his mental health problems.

**I miss the interdependence in marriage and at work, when you lose that everything falls apart. The weekends are worst. I had a dream and expectations of a good life, even in later years, which did not come true. **

#### 3.3.3. Feelings of Guilt and Shame

The older persons carried memories of previous guilt and shame, as well as in some cases more or less concealed regret about how their mental health problems had affected their family members. Guilt and shame seemed to coexist in their minds and could be suppressed or repressed to the extent that the feelings were not always consciously attributed to anything specific. The quotation below is from a woman who talked about how her depressive ill-health obliged her daughter to take on too much responsibility at an early age.

**I remember sitting on a chair waiting for the children to come home when they had left for school. I was unable to do anything, not even take care of the dishes from breakfast and put the food back into the fridge. My poor eldest daughter started at an early age to clear up when she came home from school, she had to help me over the years.**

If the older person is not encouraged to confront and discuss this kind of guilty memory, she/he has to deal with it alone. Mental barriers may subsequently be erected against one's well-being. Furthermore, the older person can indiscriminately shut out her/his family members.

Many of the participants narrated how the family was ashamed of their mental health problems, of not receiving visits when they were hospitalised in psychiatric clinics, and of not being invited to special family occasions. The families' shame influenced the older persons' own feelings of shame. There were also stories of their mental ill-health making them vulnerable and causing them to do things of which they were later ashamed.

**Finally, I found a general practitioner who would not give me up before he had found the reason for my stomach pain. I became so attached to him, we had a good dialogue and he gave me extra time. I fell in love with him and could not stay away. I wrote letters and sent him flowers. It ended with him phoning to say that he could not be my doctor anymore. How could I have believed that he would respond to my feelings? I feel that I made a fool of myself.**

## 4. Discussion 

The aim of the study was to explore experiences of family support among older persons with depressive ill-health.

*Hovering between Feelings of Belongingness and Aloneness in Relationships with Family Members*. The findings demonstrate that family support frequently involves emotional contradictions and that the older persons' dialectic hovering on a continuum between* a sense of being worthy *and* a sense of being unworthy *represents support and lack of support.

A sense of being worthy and entitled to be treated with dignity comprises internal and external dimensions related to social and cultural aspects, which are common to all human beings and at the same time unique to each person [25]. Family members could be encouraged to actively engage in preserving the older person's sense of worth and thereby her/his dignity. Anderberg et al. [25] found that the attributes of preserving dignity are individualized support, the restoration of control, respect, advocacy, and sensitive listening. These are in line with the findings of the present study, especially the longing for sensitive listening on the part of family members, something most of the participants missed.

Contradictory forces are a major part of everyday human life [26]. Erikson's [27] stages of psychosocial development might shed light on the interpretation and understanding of the older person's family support needs. A personal feeling of unity facilitates the ability to look back, evaluate, and minimise the disparity between life elements, making life meaningful or less meaningful. Basically, it involves shaping life in one's inner space [28]. As Erikson [27] argued, successfully coping with opposing attitudes, demands, and various crises is part of human growth and constitutes healthy balance. However, one's identity is never “clear-cut” or irreversible [27], and health is always a movement [29]. 

The findings revealed that unity refers to *being worthy *experienced as *being part of the family* and being capable of *living up to family expectations*. It can be assumed that the perception of being part of the family generates a sense of capability that serves as a strength in periods of suffering and ill-health. Being part of the family could be deemed mutually shared companionship as it provides a sense of belonging. A sense of being surrounded by family support is symbolised as being in the arms of the family. Supportive family relationships have an element of togetherness such as a sense of belongingness; you belong to someone and that someone belongs to you.

*A sense of being unworthy* is a weakening and diminishing form of nonsupport. For example, the older persons did not wish to be a burden on the family and anxiously questioned their ability to live up to family expectations. Such self-doubt is mentally exhausting. In the long-term it could mean increased mental tension and conflict, which can undermine health. The findings revealed that family members may not understand all aspects of suffering from long-term depressive ill-health, or as several participants stated, they were too busy with their own lives. However, it is not easy to be alone with depression [30]. Even if family members did not provide concrete support or concern, it helped if they were aware of what the older family member had to face. In view of the degree of understanding and patience that the older persons exhibited towards their family members, there were clearly no expectations on them to automatically know what to do. Relationships and connection with others are inherent in human beings, but involve risk [31]. The risk of disappointment and rejection may prevent a person from reaching out towards others, thus perpetuating feelings of loneliness. The longing for interpersonal closeness stays with every human being from infancy throughout life, and there is no one who is not threatened by its possible loss [31]. Particularly in the case of older depressed individuals, lack of family support can be an additional burden on a par with mental ill-health, which arises directly from the absence of interpersonal closeness or accessible shared relationships. The authors of this study identified both emotional and existential loneliness as well as social isolation in the findings. Loneliness can vary [31] and was detected in the older person's sense of not being worthy. Poor family relationships probably lead to loneliness and the absence of belongingness. Previous research on younger persons with mental disorders suggested that caregivers should foster a sense of belonging [32]. Martinsson et al.'s [8] study on older persons reaches a similar conclusion.

When the older depressed persons wished to be* a part of the family* and *not a burden on the family,* they often exhibited silent or explicit feelings of *guilt and shame*. Feelings of shame often lead to a sense of worthlessness [33]. According to Wiklund Gustin [34], one's self-image is that of failure for not being good enough or unable to live up to expectations, leading to a sense of strangeness. In the long term, this could mean an existential loss of family relatedness. Human beings bond through relationships. Bonding involves a reciprocal exchange between the older person and her/his family. The negative identity developed as a consequence of shame often left the older persons with a feeling of unworthiness and fear of being rejected by family members. According to this perspective, shame can prevent self-evaluation in later life. The feeling of estrangement can be a consequence of one's inability to find meaning [34]. This refers not only to the person's struggle with everyday activities, but also to the failure to live up to family expectations because of her/his condition and the wish not to become a burden. It is mainly related to the old person's existence per se and her/his attempt to fulfill the desire for relatedness and authenticity [34]. Feelings of being outside the family and rejected may prevent the older person from reaching out when she/he is ashamed. Shame and guilt are often hidden [33]; therefore it is not always easy to determine which emerged first, shame and guilt or depression. There is a movement between healthy and unhealthy shame. If, perhaps especially in old age, a person must try to adapt to extraneous expectations from others that do not correspond with her/his own principles or standards, she/he can become caught up in a spiral of unhealthy feelings: being ashamed and then ashamed of being ashamed or feeling guilty and then ashamed of the sense of guilt. Seen from the mental health perspective, this is highly distressing, even vicious. Existentially it could constitute a threat against the older person's sense of dignity. Being accepted and having one's own choices confirmed by one's family enhance a person's sense of dignity [25, 35]. On the other hand, if there is a feeling that one's personal needs are not met by family members or are subordinated to their needs, the older person will ignore or deny her/his needs, which has a negative influence on her/his dignity [35].

### 4.1. Methodological Considerations

In line with the hermeneutic approach of this study, there is always a difference between the way things seem to be and the way they really are [26, page 81]. Analysis of narrative text can be performed by using a number of techniques. This study provides one interpretation of the text; other interpretations are also possible [36]. Even though the authors' ambitions were to bracket the preunderstanding, it is never completely feasible. During the analysis the findings were thoroughly scrutinized and discussed until consensus was reached among the five authors. The study has an uneven gender sample of participants: 26 women and 3 men. The authors discussed to exclude the men. Their interviews were analysed with extra concern without finding gender specific differences. The men's experiences are included in the results. The researchers' motivation was a genuine interest in older persons with depressive ill-health and their views of family support. By citing the participants' own words, this might help the reader to judge whether or not the interpretation of the participants' experiences is credible. The findings were translated from Norwegian into English, so it was a challenge to capture the nuances in the text. Sometimes the participants also struggled to articulate and verbalise their experiences.

One reason for carrying out the present study was the lack of research on older persons' perceptions of family support. The concept of family is individual—what individuals mean when they talk or think about their own family. In this study, the participants mainly referred to their children, siblings, and partners when being interviewed about family support. The Declaration of Helsinki [24] underlines the researcher's responsibility for vulnerable individuals, and those with mental health problems are often used as an example. Although Liamputtong [37] argued against excluding groups of people in research, it is important that the researcher has some knowledge of the reasons for their vulnerability and makes adjustments based on empathy, sensitivity, and concern. In this study, the interviews were conducted by two mental health nurse researchers, which might have had a positive influence on the rich empirical material.

A focus on the family as a whole, such as individual interviews with adult children and other family members, could lead to greater insight and more detailed information and should be considered in future research.

## 5. Conclusions

The meaning of family support was feelings of belongingness versus aloneness. Family support promotes the emotional needs of older persons with depressive ill-health to be confirmed. The family plays a vital role, not always by direct assistance, but indirectly by supporting the older person*'*s own “guiding principles” for managing her/his situation. The need for family support and responsiveness in maintaining mental well-being should not be underestimated. There is a constant and deep longing for relatedness throughout life, which involves continuous, mutual balancing between the tension of giving and receiving.

The findings of this study imply that not only the older person affected by depressive ill-health but also her/his family members need support from caregivers. It is essential that the professionals are aware of what the older person needs most from her/his family in terms of her/his condition and everyday life. The older person suffering from long-term depressive ill-health should be facilitated to clarify her/his own expectations of family support. In this way, caregivers can enhance the older person's dignity, thus allowing her/him to be true and honest with her/himself. The feelings of loneliness as well as shame and guilt at poor or absent family responsiveness should be adequately addressed. Innovative nursing care can lead to improvement by focusing on acquiescence to the older person's life situation. 

## Figures and Tables

**Table 1:** Overview of the main theme, themes and subthemes.

Main theme: hovering between feelings of belongingness and aloneness in relationships with family members		

Themes	A sense of being worthy	A sense of being unworthy

Subthemes	Being part of the family	Being a burden on the family
	Being able to live up to family expectations	Being unable to live up to family expectations
		Feelings of guilt and shame
